# Ipsilateral renal dysgenesis or agenesis with tubulocystic anomalies of the mesonephric duct

**DOI:** 10.4102/sajr.v27i1.2700

**Published:** 2023-09-26

**Authors:** Anagha R. Joshi, Mridula M. Muthe, Sheethal Gonapati, Mehak R. Agarwal, Pareekshith R. Rai

**Affiliations:** 1 Department of Radiology, Lokmanya Tilak Municipal Medical College and General Hospital, Mumbai, India

**Keywords:** renal dysgenesis, renal agenesis, tubulocystic anomaly, mesonephric duct, ectopic insertion

## Abstract

Tubulocystic anomalies of the mesonephric duct (MND) are a rare group of related entities with a perplexing clinical presentation. Ultrasound is a useful screening investigation, which can help identify a dysplastic kidney or point to renal agenesis and identify cystic or tubulocystic changes in the structures derived from the ureteric bud or MND. Further evaluation with MRI can help in characterisation and direct management. The presence of a dysplastic kidney or absence of a kidney should prompt further evaluation for associated ureteric abnormalities such as ectopic insertion or ureterocoele, which could lead to a diagnosis of tubulocystic anomaly of the MND.

**Contribution:**

The authors describe two cases, briefly outline the diagnostic approach and summarise the literature on management. Reporting radiologists should be aware of these entities.

## Introduction

Congenital abnormalities of the excretory system are commonly encountered and have a wide spectrum of clinical presentation. Of these, tubulocystic anomalies of the mesonephric duct (MND) are quite infrequent. Understanding the relevant embryology and presentation of such anomalies is crucial for accurate diagnosis and decisions on appropriate management.

Embryologically, the kidneys and urinary tract develop from the intermediate mesoderm and cloaca. The kidneys have three sets of precursors: the pronephros, mesonephros and metanephros. The pronephros is a transient, non-functioning precursor, which spontaneously regresses. The pronephros elongates caudally to fuse with the cloaca to form the pronephric duct, which in turn is the precursor of the MND. The mesonephric (Wolffian) and paramesonephric (Mullerian) ducts are paired embryonic structures that are precursors of the male and female internal genitalia, respectively. In addition, the MNDs are involved in the development of the kidneys and the trigone of the bladder in both genders.

Functioning tubules that excrete urine develop from the mesonephros but these regress as well. In males, the mesonephric tubules are the precursors of the epididymal ducts and the efferent ducts. The ureteric bud develops from the caudal portion of the MND and branches into the metanephric mesenchyme to form the metanephros, which is the immediate predecessor of the adult functioning kidney. Reciprocal interactions between the ureteric bud and the metanephric mesenchyme result in elongation and branching of the ureteric bud to form the collecting system and transform the metanephric mesenchyme into primitive nephrons.^1,2,3^

The developing foetus is initially ambisexual and both genders have a common sequence of development with subsequent differentiation being determined by the internal genitalia. Female internal genitalia will develop unless a testis is present. The Sertoli cells of the foetal testis produce Mullerian Inhibiting Factor (or Anti-Mullerian Hormone, AMH) which impedes the development of the Mullerian duct while the testosterone produced by the Leydig cells stimulates the further differentiation of the MND into the vas deferens, epididymis and seminal vesicles. In the absence of Mullerian Inhibiting Factor, paramesonephric ducts differentiate into the fallopian tubes, the uterus and the upper portion of the vagina.^3^ It is believed that the absence of androgenic stimulation is responsible for the involution of the MNDs in females.^4,5^ However, these degenerated ducts do leave behind certain vestigial remnants. In males, the appendix of the testis and the utriculus prostaticus are remnants of the paramesonephric duct.^3^ In females, the commonly described remnants include Gartner’s ducts, epoophoron, paraoophoron and the appendix vesiculosa.^4^ The Gartner’s ducts are paired ducts that course lateral to the vagina in the broad ligament and are the remnants of the caudal portion of the MND. Obstruction of these ducts can give rise to Gartner’s duct cysts. Epoophoron is analogous to the male epididymis and they are generally located in the lateral portion of the broad ligament between the ovary and fallopian tubes. Paraoophoron is analogous to the male paradidymis and is located in the medial part of the broad ligament. Certain rarely described pathologies that are related to MND remnants in females include para-ovarian cysts, mesonephric hyperplasia, mesonephric carcinoma and Female Adnexal Tumours of probable Wolffian origin (FATWO).^5^

Reciprocal interactions between the ureteric bud and the metanephric mesenchyme are essential, as the development of one cannot progress without the other; that is, the ureteric bud fails to develop in the absence of metanephric mesenchyme and vice versa.^2^ Renal agenesis as well as renal dysplasia occur when these interactions are interrupted. Early division of the ureteric bud leads to formation of duplication abnormalities.^3^ Defective incorporation of the ureteric bud with the bladder may result in cystic abnormalities of the terminal ureter such as ureterocoele or ectopic insertion into the urogenital sinus.^6^ Tubulocystic anomalies of the MND occur because of disruption in the interactions between the metanephric mesenchyme and the ureteric bud.^7^

**FIGURE 1:**
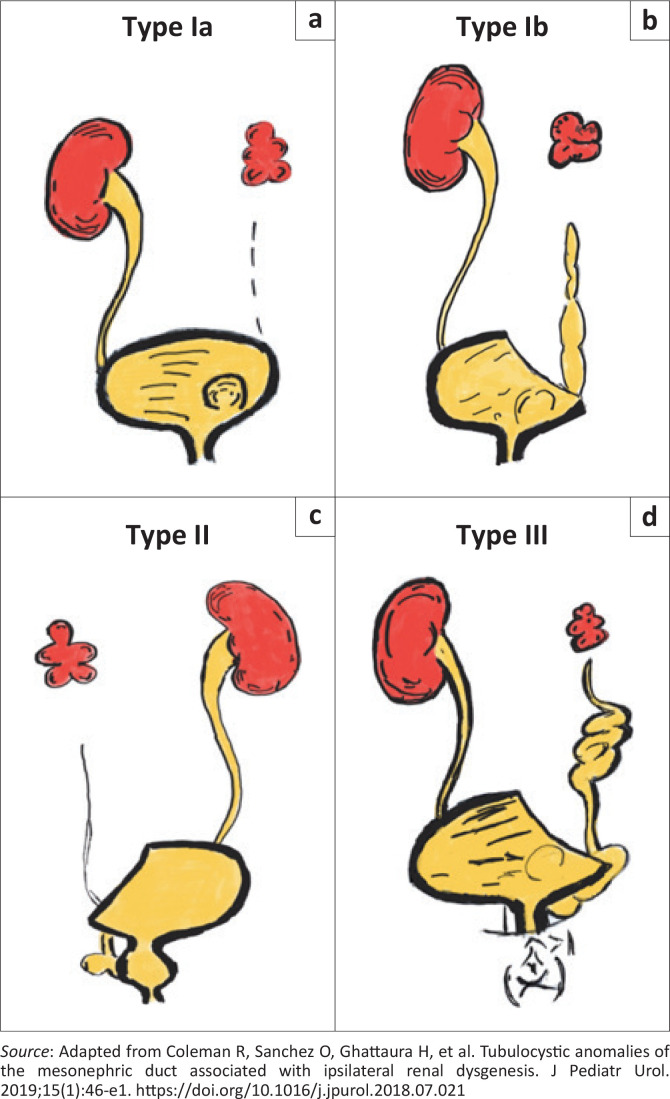
Classification of tubulocystic anomalies of the mesonephric duct. (a) Isolated ureterocoele. (b) Ureterocoele with tubulocystic changes in the remnant ureter. Note that Type I anomalies are characterised by orthotopic insertion of the ureteric bud structures into the superolateral aspect of the trigone of the urinary bladder. (c) Ureteric bud structures are seen inserting ectopically into the vagina, urethra, et cetera. (d) Complex anomalies with remnants from the ureteric bud and the mesonephric duct, includes anomalies such as obstructed hemivagina, ipsilateral renal agenesis (OHVIRA) and Zinner syndrome as elaborated in the text.

Coleman et al. conducted a single centre retrospective cohort study and analysed the findings in 19 patients who presented over a 7-year period and broadly classified them into three types as described in the next section.^8^ These are further illustrated in Figure 1.

### Classification

Type I – These are characterised by orthotopic insertion of ureteric bud structures (ureter and trigone):

- Type Ia – Orthotopic insertion of the ureter with an associated isolated ureterocoele.
- Type Ib – Orthotopic insertion of the ureter with a terminal ureterocoele and tubulocystic changes in the remnant ureter.

Type II – Remnant structures derived from the ureteric bud, which are seen inserting ectopically into structures such as the urethra, bladder neck, vagina, uterus, etc.

Type III – Complex anomalies with remnant structures derived from the ureteric bud and the MND. These arise from the MND cranial to the ureteric bud aside from the ureteric bud itself.

This includes anomalies such as Zinner syndrome, which is characterised by a triad of renal agenesis, seminal vesicle cysts and ejaculatory duct obstruction. Abnormal development of the distal part of the ureteric bud results in atresia of the ejaculatory duct with resultant formation of seminal vesicle cysts while dysplasia of the proximal ureteric buds results in renal agenesis or dysplasia.^9^

Obstructed hemivagina, ipsilateral renal agenesis (OHVIRA) syndrome also known as Herlyn–Werner–Wunderlich (HWW) syndrome is also considered a type III anomaly. It is characterised by anomalies of the Mullerian and MNDs. Mullerian duct anomalies that are seen include uterine didelphys and obstructed hemivagina. Classically, it is associated with renal agenesis but other anomalies such as dysplastic kidneys, cross-fused ectopia and duplicated kidneys have been described. Gartner duct cysts and pelvic endometriosis may also be seen.^10^

This report describes two paediatric cases with type II abnormalities, that is, with ectopic insertion of the ureter.

## Case presentations

### Case 1

**FIGURE 2:**
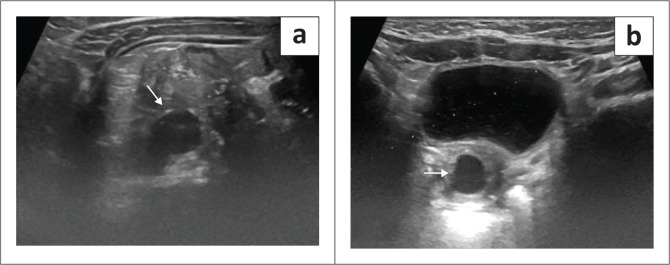
(a) Transverse ultrasound image revealed an ectopic dysplastic right kidney (*white arrow*) in the umbilical region. (b) Transverse ultrasound image showed a focal cystic dilatation of the right tubulocystic ureter posterior to the bladder (white arrow). The insertion of ureter was not well visualised.

A 6-month-old female presented with complaints of continuous dribbling of urine from the vagina and occasional fevers as noticed by her mother. An ultrasound of the abdomen and pelvis was performed as the initial imaging examination and revealed an empty right renal fossa. An ectopic dysplastic kidney with cystic areas was seen to the right of the umbilical region. A dilated and tortuous right ureter was identified distal to the kidney but its insertion was not well appreciated on ultrasound (Figure 2). The left kidney was normal in position, outline and morphology, and the left ureter was undilated. Static MR urography was advised for further assessment and for evaluation of the right ureter.

**FIGURE 3:**
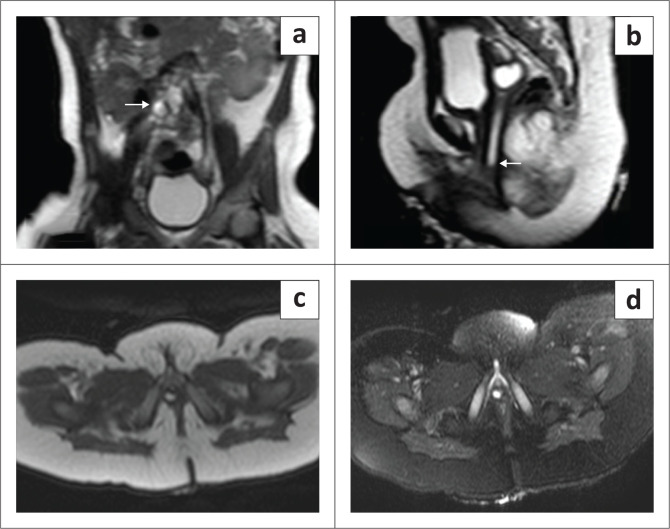
(a) T2-coronal image indicated an ectopic dysplastic right kidney with a few non-communicating cysts (white arrow) in the umbilical region. (b) T2 sagittal image shows a tortuous and dilated right ureter (white arrow), which is coursing well below the level of the bladder neck. (c) T2 axial non-fat-sat and (d) fat-sat images depicted the ectopic insertion of the right ureter into the posterolateral aspect of the vagina.

Static MR urography (Figure 3a and b) confirmed an empty right renal fossa. A dysplastic ectopic right kidney with a few non-communicating cysts was seen in the paramedian right lumbar region. A dilated, tortuous right ureter was identified and was seen coursing posterior to the urinary bladder with an ectopic insertion into the posterolateral aspect of the vagina (Figure 3c and d). The patient underwent excision of the ectopic ureter and was doing well on follow-up.

### Case 2

**FIGURE 4:**
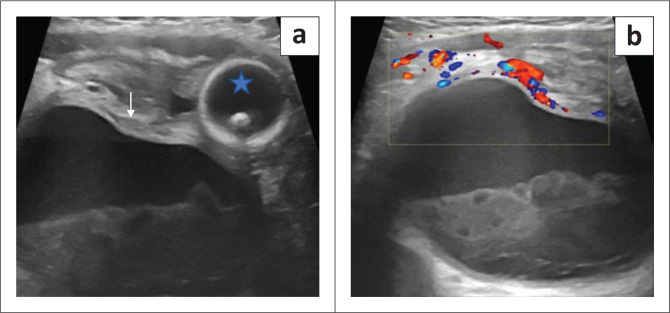
(a) Transverse grey scale and (b) colour doppler ultrasound images demonstrated a well-defined, anechoic, cystic lesion (*white arrow*) with dependent debris. The urinary bladder with a foley’s catheter bulb (star) is seen separately.

A 7-month-old male presented with abdominal distension. No history of crying spells during micturition or dribbling of urine was noticed. The mother provided a history of agenesis of the right kidney on antenatal ultrasound, which was confirmed on postnatal ultrasound. Additionally, at 6 months of age, the patient underwent bilateral orchidopexy for undescended testes. A routine ultrasound scan (Figure 4a and b) performed at that time revealed a well-defined anechoic cystic lesion with dependent debris in the right hemipelvis. The urinary bladder was displaced to the left and was seen separately. The left kidney was normal in position, outline and morphology, and the left ureter was undilated. Static MR urography was advised for further evaluation and diagnosis.

**FIGURE 5:**
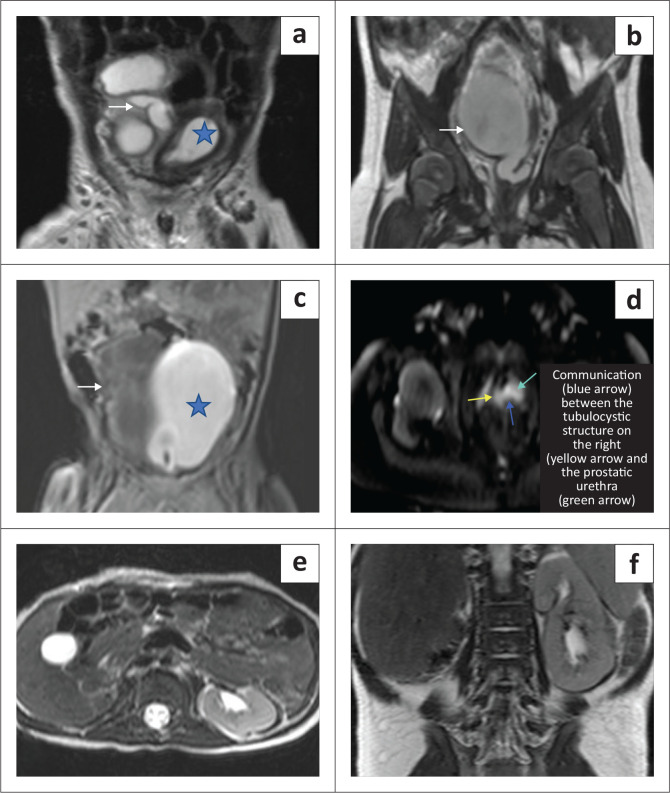
(a) T2 coronal image showed a tubular convoluted cystic structure (*white arrow*) in the right paramedian abdomino-pelvic cavity, representing tubulocystic changes in the right ureter. The urinary bladder was visualised on the left side of pelvis (*star*). (b) T2 coronal image showed a large, well-defined, tubular, cystic structure in the pelvic midline, which demonstrated a suspicious communication with the prostatic urethra inferiorly. (c) T1 coronal (post-retrograde contrast administration) image showed the contrast filled, displaced urinary bladder on the left side of pelvis. The cystic lesion on the right did not opacify with contrast (*white arrow*). (d) T2 fat-sat axial image depicted the communication between the tubulocystic structure on the right and the urethra. (e) Axial and (f) coronal T2 images revealed an absent right kidney.

Static MR urography (Figure 5a–d) showed a large, T2 hyperintense well-defined, tubular, fluid-filled structure with T2 hypointense dependent debris in the midline and right paramedian pelvis. This structure was seen to be communicating with the prostatic urethra inferiorly. The urinary bladder was visualised separately and was displaced to the left. An empty right renal fossa was appreciated (Figure 5e and f).

On the subsequent retrograde urethrogram contrast study (using 2 mL gadolinium mixed with saline), there was no leakage of contrast into this structure. In view of its large size and as per the parent’s wishes, ureteric excision was performed and the patient was doing well on follow-up.

## Discussion

While the different types of tubulocystic anomalies may present variably, when imaging these patients it is important to thoroughly evaluate the entire genitourinary system.Ultrasound can be used as an initial investigation to assess renal morphology and position, exclude urinary duplication anomalies, evaluate ureteric insertion sites, exclude a ureterocoele and rule out other associated anomalies related to the MND.

Evaluation with MRI is the gold standard investigation when assessing the anatomy of the kidney and the collecting systems. It is useful in the identification of a ureterocoele and also aids in identifying the site of insertion of an ectopic ureter. It is also useful in the evaluation of other MND anomalies such as seminal vesicle cysts.^11^

Nuclear scintigraphy is primarily used to assess residual renal function and exclude scarring and complications such as pyelonephritis. When used in conjunction with other imaging modalities, it is useful to diagnose multicystic dyplastic kidney (MCDK) disease, although its routine use is not recommended.^12^

Coleman et al.^8^ noticed that most patients (*n* = 11) in their study only required conservative management. Only patients with a large ureterocoele or with bladder neck compression required endoscopic puncture to facilitate drainage. The MCDK generally involutes partially or completely with time but tubulocystic remnant ureteric bud structures tend to persist. Some of these patients required surgery for associated anomalies such as hypospadias, hemivaginal septum incision (in OHVIRA) and epididymal cyst excision. Certain anomalies such as congenital absence of the vas deferens, seminal vesicle cysts, uterine or vaginal anomalies may be difficult to detect in the pre-pubertal age group, which highlights the need for sequential follow-up imaging.

Intervention is only indicated in the presence of large ureterocoeles or if symptomatic from obstruction of urine outflow, pain or recurrent infection. Transurethral puncture drainage is safe in these cases and leads to symptom resolution. Exteriorisation of perineal or vulval cysts is not recommended as there may be secondary infection or they may result in a chronically discharging sinus. Thus, most patients only require conservative management as secondary infection is rare and prophylactic antibiotics are often unnecessary. Moreover, any surgery to excise these tubulocystic anomalies carries an inherent risk of damaging the adjacent derivatives of the MND or the bladder neck and the continence mechanism as they have a fairly complex anatomy making complete resection very difficult. Management decisions should, therefore, be made on a case-by-case basis after considering the possible morbidity associated with surgery. For those on conservative management, rigorous surveillance imaging is adequate.^8^

## Conclusion

Tubulocystic anomalies of the MND are very rare. In the absence of classic clinical presentation, multimodality imaging plays a crucial role in their diagnosis, aiding in early detection and ensuring appropriate management. Ultrasound can be considered as the initial modality of choice while MRI is necessary for confirmation and further detailed characterisation. The aforementioned cases highlight the need for the reporting radiologist to keep this possibility in mind when evaluating paediatric congenital renal anomalies.
